# Involvement of Nucleotide Excision Repair and Rec-Dependent Pathway Genes for UV Radiation Resistance in *Deinococcus irradiatisoli* 17bor-2

**DOI:** 10.3390/genes14091803

**Published:** 2023-09-15

**Authors:** Gayathri Subramani, Sathiyaraj Srinivasan

**Affiliations:** Department of Bio & Environmental Technology, College of Natural Science, Seoul Women’s University, Seoul 01797, Republic of Korea; gaya.micro@gmail.com

**Keywords:** *Deinococcus*, radiation resistance, γ-radiation, radiation resistance, nucleotide excision repair, PacBio RS II, complete genome

## Abstract

Strain *Deinococcus irradiatisoli* 17bor-2 was isolated from a soil sample exposed to γ radiation at Seoul Women’s University, Republic of Korea. The genus *Deinococcus* is a Gram-negative, coccus-shaped, and extremophilic bacterium, well renowned as being a radiation-resistant bacterium. Therefore, the mechanism behind the resistance to radiation and the gene responsible for the resistance could be helpful for detailed experimental studies with biotechnological applications. To study the involvement of genes in UV radiation resistance in strain 17bor-2, the genomic DNA of the strain was sequenced and constructed using the Pacific Biosciences RS II system. In addition, the complete genome sequence of strain 17bor-2 was annotated and interpreted using the Genomes–Expert Review (IMG-ER) system, along with Prodigal and JGI GenePRIMP analysis. The genome analysis of strain 17bor-2 revealed evidence of excinuclease UvrABC genes, which are key enzymes in the nucleotide excision repair (NER) mechanism, as well as genes from the recA-dependent and recQ pathways. The genome of strain *Deinococcus irradiatisoli* 17bor-2 was a circular chromosome comprising 3,052,043 bp with a GC content of 67.0%, including 2911 coding sequences (CDs), 49 tRNA genes, and 9 rRNA genes. In addition, their complete genome sequence annotation features provided evidence that radiation resistance genes play a central part in adaptation against extreme environmental conditions. In recent decades, excision repair genes have been indicated in considerable detail for both prokaryote and eukaryote resistance against UV-C radiation.

## 1. Introduction

Ultraviolet radiation (UV-C) is a potent agent in the eradication of bacterial cells, primarily through the destruction of their DNA. This form of radiation instigates a reaction between two molecules of thymine, resulting in the creation of very stable thymine dimers [1]. Ionizing radiation impairs DNA and other cell macromolecules, causing cellular damage [2,3,4]. However, extremophiles are gifted with a self-repair mechanism that can be activated by exposure to radiation. *Deinococcus radiodurans*, a Gram-positive extremophilic bacterium, is a remarkable example of such an organism, showcasing an impressive resistance to a wide array of stressors, including ionizing radiation, desiccation, UV radiation, and oxidizing agents. The evolutionary trajectory of the *Deinococcus* species is genuinely remarkable. Over time, these organisms have developed the ability to not only grow consistently at a rate of six kilorads (60 Gy)/h but also to endure sudden exposures to γ radiation levels that surpass 1500 kilorads. This unique adaptation showcases their resilience and ability to thrive in conditions that would be lethal to many other organisms. Their capacity to withstand such high radiation levels offers intriguing insights into the potential mechanisms and evolutionary pressures that have shaped their survival strategies. Microorganisms are widely utilized in evolutionary experimental models. Researchers have started expanding *Deinococcus* spp. in biotechnology and bioremediation fields in recent years due to their specific ability to grow under high radiation conditions and their novel engineered functions. The phenomenon of radiation resistance, which has been found in particular organisms, can be ascribed to four fundamental mechanisms. Firstly, protein oxidation mitigation [5] is employed to maintain the structural integrity of biological proteins. Additionally, the advent of novel DNA repair mechanisms [6] offers a more practical approach to repairing DNA damage caused by radiation. One additional issue that should be considered is the condensation of the nucleoid structure [7], which potentially provides a safeguard for the genetic material. Finally, the optimization and modification of known DNA repair enzymes are undertaken to boost their efficacy in mitigating radiation-induced damage [8]. Moreover, it is important to highlight that genetic breakthroughs and evolutions in the existing DNA repair mechanisms can play a crucial role. Genetic changes of this form can exert a substantial impact on an organism’s capacity to establish a phenotype that is resistant to ionizing radiation [8]. This highlights the dynamic character of evolutionary adaptations in response to environmental pressures.

As our understanding of the complex processes involved in DNA repair continues to expand, so does our appreciation of the sophisticated strategies that organisms employ to resist potentially lethal environmental insults. Among these strategies, both the nucleotide excision repair (NER) pathway and the Rec-dependent recombination pathway have emerged as vital systems in repairing DNA damage caused by various factors, including ultraviolet (UV) radiation [9,10]. UV radiation, a ubiquitous environmental stressor, imposes significant damage to cellular DNA, particularly in the form of cyclobutane pyrimidine dimers (CPDs) and 6-4 photoproducts (6-4 PPs) [11,12,13]. Organisms’ ability to survive and thrive in environments with high UV radiation levels depends mainly on their ability to efficiently repair this UV-induced DNA damage [14,15].

The NER pathway, which is highly conserved among organisms from bacteria to humans, plays a crucial role in this context [16]. NER (nucleotide excision repair) primarily focuses on fixing severe DNA distortions that disrupt the helical structure of the molecule, including those produced by UV radiation. This repair pathway comprises two sub-pathways: global genomic NER (GG-NER) and transcription-coupled NER (TC-NER) [17]. GG-NER operates throughout the genome, scanning for and repairing DNA lesions, while TC-NER preferentially repairs transcribed DNA strands of active genes [18]. The elaborate coordination of multiple protein components characterizes the NER pathway, with each component performing a specific function in damage recognition, incision, removal, and DNA synthesis [19]. In addition to NER, the Rec-dependent recombination pathway, named for the RecA protein in bacteria and its functional homologs Rad51 and Dmc1 in eukaryotes, is instrumental in maintaining genomic integrity following DNA damage [20]. This pathway becomes particularly relevant when the DNA replication machinery encounters a lesion that it cannot bypass, leading to replication fork stalling. In such cases, Rec-dependent recombination aids in repairing the lesion and restarting the stalled replication fork, thereby confirming the completion of DNA replication [21,22]. Understanding the intricate interplay of NER and Rec-dependent pathways in UV resistance is not only fascinating from a fundamental science perspective but also has significant implications for various fields. For instance, the knowledge gained from this research can provide insights into the mechanisms underlying human genetic disorders associated with defects in these pathways. Additionally, it may shed light on the development of microbial resistance to UV disinfection, an issue of increasing concern in the era of antibiotic resistance.

Given the importance of these repair pathways, this review aims to comprehensively explore the role and interplay of NER and Rec-dependent pathway genes in UV radiation resistance. We will delve into the molecular mechanisms of these pathways, discussing the functions of their key components and how they cooperate to mitigate the harmful effects of UV radiation. Further, we will highlight the current state of knowledge regarding their regulation and coordination. Finally, we will consider the broader implications of these findings for human health and disease, as well as their potential applications in biotechnology and environmental science. In summary, this genomic analysis will provide an in-depth analysis of how strain 17bor7 harnesses the power of NER and Rec-dependent pathway genes to combat the deleterious effects of UV radiation. We hope that this serves as a valuable resource for researchers and scholars interested in the fields of DNA repair, UV radiation biology, human genetic disorders, microbiology, and environmental science.

The in-depth analysis of the genomes of radiation-resistant bacteria, like *Deinococcus*, holds significant implications. Not only does it shed light on their unique survival mechanisms, but it also reveals that some DNA repair genes have been further characterised, offering more profound insights into their roles and functions. Understanding these genes can provide valuable information on the intricate processes that enable such bacteria to thrive in high-radiation environments [22]. In addition to their noteworthy capacity for radiation resistance, *Deinococcus* spp. exhibit additional outstanding characteristics. The ability of these organisms to degrade solvents such as toluene and metabolise sugars and sugar polymers makes them a highly interesting prospect in the field of industrial biotechnology. The broad spectrum of applications in the sector is underscored by the multifarious skills of these organisms. These characteristics inspired us to sequence the genome of *strain* 17bor-2. Here, we present the complete genome sequence of strain 17bor-2 to find survival strategies after γ irradiation. Strain 17bor-2 was isolated from a soil sample collected at Seoul Women’s University, Republic of Korea (37°62′79.44″ N, 127°09′04.85″ E), and the complete genome sequence was reported.

## 2. Materials and Methods

### 2.1. Culturing and Radiation Resistance Analysis

Approximately 10^9^ CFU/mL of bacterial samples were cultured in TGY broth containing 1% tryptone, 0.1% glucose, and 0.5% yeast extract (obtained from Difco Laboratories, Detroit, MI, USA). The primary objective was to examine the survival trajectory of these bacteria following γ and UV-C radiation exposure. For the investigation, bacteria in their stationary growth phase were chosen to ensure accurate results. The exposure procedure utilized a cobalt-60-based γ irradiator that delivered radiation at a 70 Gy/min dose and a radiation intensity of approximately 100 kCi (3.7 PBq). *D. radiodurans* R1^T^ (DSM 20539^T^) and *E. coli* K12 (KCTC 1116) were used as positive and negative control strains, respectively, for comparative analysis. Bacterial samples were diluted and deposited on microplates following radiation treatment. In order to maintain uniformity and consistency, the diluted samples were subsequently plated in duplicate on TGY agar plates, as suggested in prior studies [23,24,25].

### 2.2. Genome Sequence Project

The total genomic deoxyribonucleic acid (DNA) was isolated using the DNA extraction kit manufactured by Solgent. The extraction procedure was conducted in accordance with the manufacturer’s established protocols to maintain the highest level of genomic material integrity and purity. Subsequently, the genomic sequence of the strain designated as 17bor-2 deposited at DDBJ/EMBL/GenBank can be accessed using the accession number CP029494. The construction of the genome DNA library and the subsequent sequencing process were effectively performed utilizing the Pacific Biosciences RS II sequencing platform. Table 1 provides a systematic presentation of comprehensive details and specific information pertaining to the genome sequencing project with the association of Minimum Information about a Genome Sequence (MIGS) version 2.0 identifiers [26].

### 2.3. Library Construction and Assembly

A library was constructed precisely according to the Pacific Biosciences RS II sequencing method manual. Then, the obtained 145,890 sequencing reads of strain 17bor-2 were assembled using PacBio SMRT Analysis (version 2.3.0) with default options. The final assembly resulted in 1 contig generating a corresponding genome size of 3,052,043 (Table 2).

### 2.4. Genome Annotation

The comprehensive functional annotation and gene prediction for strain 17bor-2 were meticulously carried out utilizing the Integrated Microbial Genomes–Expert Review (IMG-ER) platform. This was complemented by the use of Prodigal software v. 2.6.3 and the JGI GenePRIMP pipeline [27]. For the identification of tRNA genes within the genome, the specialized tRNAScan-SE tool was employed [28]. In the quest to predict ribosomal RNA genes and other non-coding RNAs (ncRNAs), tools such as RNAmmer_ENREF_39 [29] and Infernal [30] were employed. The process of pinpointing protein-coding genes was initiated using Prodigal software. This initial identification was further refined through a rigorous round of manual curation facilitated by the expertise provided by the JGI GenePRIMP pipeline. Subsequent to these processes, the predicted coding DNA sequences (CDSs) underwent a thorough search against renowned protein databases. This involved cross-referencing with the TIGR-fam, Pfam, and COG databases, whereby all of which are seamlessly integrated within the IMG systems. This multi-faceted approach ensured a holistic and accurate representation of the genomic and functional landscape of strain 17bor-2.

## 3. Results

### 3.1. Morphology and Radiation Resistance Analysis

The bacterial strain 17bor-2 was cultured from a soil sample obtained at Seoul Women’s University, Republic of Korea (GPS; 37°62′79.44″ N, 127°09′04.85″ E). The bacterial strain 17bor-2T exhibits characteristics of being Gram-negative and possesses pale-yellow pigmentation. Morphologically, it is observed as a short-rod-shaped bacterium. When subjected to γ and UV-C radiation, the D10 value (the dose required to reduce the population by 90%) for strain 17bor-2 is measured at 4 kGy and 400 J/m^2^, respectively. In contrast, *Deinococcus radiodurans* R1 demonstrates a significantly higher radiation resistance with D10 values exceeding 12 kGy for γ radiation and 700 J/m^2^ for UV-C radiation.

### 3.2. Genomic Properties

**Figure 1:**
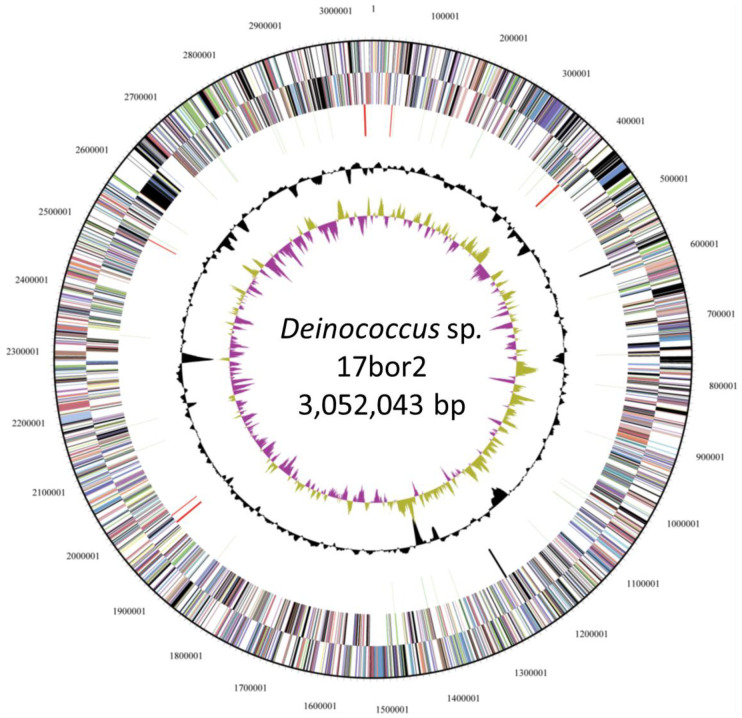
Graphical circular map of *Deinococcus* irradiatisoli17bor-2. From the outside of the map to the inside: color by COG categories and RNAs on the forward strand, color by COG categories and RNAs on the opposite strand, color by GC content and GC skew, and color by genes on the forward strand and genes on the reverse strand.

The complete genome of strain 17bor-2 consists of a circular chromosome of 3,052,043 bp with a GC content of 67.0%. No plasmid was found in strain 17bor-2; the presence of plasmid can vary in *Deinococcus* isolates: some isolates harbor plasmids, while others do not [31,32]. The presence or absence of plasmids may be influenced by factors such as the isolation source, environmental conditions, and the specific strain’s genetic makeup. Upon thorough analysis of the complete genome sequence, a total of 2911 genes were identified. This included 49 tRNA genes and nine rRNA genes. Out of these, 2003 genes were annotated and were assumed to possess putative functions. The genes that were not assigned a definitive function were categorized as either hypothetical or were designated as converted hypothetical proteins. Furthermore, a subset of 1535 genes was systematically grouped into 25 distinct COGs (Clusters of Orthologous Groups) [33]. The detailed distribution and classification of these genes within the COGs can be visualized in Figure 1 and are tabulated in Table 3.

### 3.3. Perceptions from the Genome Sequence

The genomic analysis of strain 17bor-2 warranted a thorough examination of its genetic makeup, revealing the existence of a distinct gene cluster that holds significant importance in the process of nucleotide excision repair (NER). The NER pathway encompasses the genes in charge of encoding the protein complex referred to as exonuclease UvrABC. Furthermore, the genetic components encompassed in this study comprise the recA-dependent and recQ pathways, which play crucial roles in the repair of DNA lesions induced by irradiation. Further exploring the operational characteristics of the exonuclease UvrABC reveals its composition consisting of several subunits, and among which subunits A and C play a crucial role in the nucleotide excision repair (NER) pathway. The UvrABC exonuclease complex exhibits a remarkable capacity to identify and acknowledge DNA structural modifications resulting from damage induced by ultraviolet (UV) radiation. Upon identification, the complex initiates an active process of repairing the site of damaged DNA [34].

The bacterial strain 17bor-2 harbors a genomic sequence that contains the genetic information necessary for the production of the UV damage repair endonuclease, known as UvdE. Interestingly, the amino acid sequence of the encoded UvdE demonstrates a striking similarity of 86% to the UVDE protein identified in *D. radiodurans* R1 [22]. The observed similarity in the sequence between UvdE in strain 17bor-2 and its counterpart in *D. radiodurans* R1 implies that UvdE in strain 17bor-2 likely plays a crucial role in conferring strong resistance to UV radiation, similar to its counterpart in *D. radiodurans* R1. The strain 17bor-2 genome was compared to *D. radiodurans* R1^T^ and *Deinococcus geothermalis* DSM 11300^T^; one explanation for this observation is the existence of shared biology between members of these genera (Table 4).

## 4. Discussion

The complete genome analysis and thorough annotation of the novel bacterial strain 17bor-2 have unearthed the vital genes responsible for the DNA recombination repair pathways. The genome of *D. irradiatisoli* 17bor-2 was discovered to encode the protein complex excinuclease UvrABC, along with recA-dependent and recQ pathways. These components are integral in promoting DNA repair, thereby contributing significantly to the extreme radiation resistance characteristic of the bacteria [8]. UvrA proteins play a crucial role in the nucleotide excision repair (NER) pathway [35]. This pathway is tasked with the restoration of an array of structurally diverse lesions, particularly those induced by ultraviolet light (UV) [36]. The mechanism through which the NER pathway operates involves the recognition and excision of the damaged DNA segment in a process known as dual-incision. In the context of *D. radiodurans*, if there is a deficiency in UvrA, the normal function can be entirely restored by expressing *E. coli* UvrA in the mutant *D. radiodurans* [37,38]. In stark contrast to UvrA, the recA gene in *D. radiodurans* has been found to occupy a unique position of significance in the phenotype of extreme radiation resistance. Mutations in the RecA gene render the bacteria susceptible to both ionizing radiation and UV. Strikingly, the introduction of *E. coli* RecA in *D. radiodurans* is unable to compensate for the RecA deficiency, suggesting a unique role of *D. radiodurans* recA in providing radiation resistance [38].

The UvrABC pathway, encoded by the genome of *D. irradiatisoli* 17bor-2, is essentially an excinuclease complex composed of three distinct proteins: UvrA, UvrB, and UvrC. Within the nucleotide excision repair (NER) pathway, the UvrA protein initially recognizes and binds to the damaged DNA site [36]. Subsequently, it recruits UvrB to the damaged site and detaches itself, leaving UvrB bound to the DNA. UvrB, in association with UvrC, facilitates a dual-incision event, removing the damaged DNA segment. Subsequent to the formation of the DNA gap, it is addressed and filled by the enzyme DNA polymerase. After that, the small rupture, or nick, in the DNA is effectively sealed by DNA ligase, ensuring the restoration and preservation of the DNA molecule’s integrity [39]. Interestingly, the 17bor-2 strain can compensate for a UvrA deficiency by expressing the *E.coli* UvrA, implying that the functions of UvrA in these two organisms might be conserved and interchangeable [38]. This adaptability showcases the resilience of *D. irradiatisoli* 17bor-2 and its flexibility to overcome DNA damage.

When discussing DNA damage, it is essential to note that this phenomenon is not just limited to radiation-induced lesions. Various environmental factors such as chemical mutagens, oxidative stress, and replication errors can cause DNA damage. In this context, the role of DNA repair pathways like NER (with its UvrABC excinuclease) and homologous recombination (with its key player recA) is indispensable for maintaining genomic integrity [40]. As previously discussed, the function of UvrA in the NER pathway and recA in homologous recombination seems to be conserved among bacteria, which indicates an evolutionarily established mechanism for combating DNA damage [36,38]. The interplay between these DNA repair pathways contributes to the robustness of the radiation resistance mechanism. Multiple DNA repair pathways can recognize and repair a single DNA lesion. This redundancy provides backup mechanisms, contributing to the overall resilience of *D. irradiatisoli* 17bor-2 and other radioresistant organisms. Studying these processes provides a valuable framework for understanding the molecular mechanisms underpinning radiation resistance. It is this blend of individual genes, their interactions, and the overall genetic network that propel these bacteria to survive and thrive under extreme conditions.

## 5. Conclusions

This comprehensive study presents an in-depth investigation of *D. irradiatisoli* 17bor-2, a bacterium showcasing impressive resistance towards UV and γ radiation. The bacterium was isolated from soil samples collected from the Seoul Women’s University campus in the Republic of Korea. The unique qualities of this strain, such as its adaptability to extreme environmental conditions and robust radiation resistance, lend itself to significant scientific interest. Detailed genome sequencing of *D. irradiatisoli* 17bor-2 revealed fascinating insights into its survival strategy in hostile environments. Consistent with other radiation-resistant bacteria, this strain encodes several crucial enzymes instrumental in DNA repair. Among these are key components of the bacterial RecA-dependent pathway and the UvrABC pathway [36]. The UvrABC pathway, in particular, is a part of the nucleotide excision repair (NER) system, a crucial mechanism that recognizes and repairs a broad range of structurally diverse DNA lesions [39]. This pathway allows *D. irradiatisoli* 17bor-2 to combat UV-induced DNA damage effectively, further enhancing its survival in radiation-rich environments. In addition, the recA gene plays a pivotal role in homologous recombination, a process fundamental for maintaining genomic stability. The recA gene facilitates the formation of striated filaments on both single-stranded and double-stranded DNA, promoting efficient DNA strand exchange. Moreover, it exhibits DNA-dependent nucleoside triphosphatase activity [41]. Given these findings, it is clear that the genome of the *Deinococcus* strain 17bor-2 provides invaluable information regarding radiation resistance potential against a range of radiations, including γ rays, X-rays, UV, and radio waves. The robust DNA repair mechanisms and interplay between various gene pathways are the cornerstones of this bacterium’s remarkable resilience.

In conclusion, the comprehensive genomic analysis of *D. irradiatisoli* 17bor-2 has illuminated the intricate molecular mechanisms that these microorganisms harness to survive and thrive in extreme environments. This knowledge could pave the way for various applications, from improving the efficacy of cancer radiation therapy to developing radiation-resistant materials for space exploration. Moreover, the potential bioremediation applications of such radioresistant bacteria could offer innovative solutions to manage radioactive waste in contaminated environments.

## Figures and Tables

**Table 1:** Genome sequencing project information. * MIGS: Minimum Information about a Genome Sequence.

MIGS * ID	Property	Term
MIGS-31	Finishing quality	Finished
MIGS-28	Libraries used	PacBio library
MIGS-29	Sequencing platforms	Pacific Biosciences RS II
MIGS-31.2	Sequencing coverage	156×
MIGS-30	Assemblers	PacBio SMRT Analysis 2.3.0
	NCBI accession	CP029494
	GOLD ID	Ga0307136
	NCBI bioproject ID	PRJNA471975
MIGS-13	Source material identifier	17bor-2

**Table 2:** Genome statistics. ^a^ The cumulative value was determined by either the total length of the genome measured in base pairs or by the aggregate count of protein-coding genes present within the annotated genomic sequence. Abbreviations: bp, base pair; DNA, deoxyribonucleic acid; RNA, ribonucleic acid.

Attribute	Value	% of Total ^a^
Genome size (bp)	3,052,043	100.0
DNA-coding region (bp)	2,492,501	88.4
DNA G + C content (bp)	2,112,710	67.0
No. of contigs	1	100.0
Total genes	2911	100.0
rRNA genes	9	0.1
tRNA	49	1.7
Protein-coding genes	2128	97.2
Pseudo genes	0	0.0
Genes with function prediction	2003	83.0
Genes assigned to COGs	1535	72.4
Genes assigned Pfam domains	1421	83.6
Genes with signal peptides	306	9.6
Genes with transmembrane helices	824	23.0

**Table 3:** The number of genes associated with COG functional categories.

Code	Value	%	COG Color *	Description
J	141	6	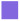	Translation
A	0	-	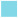	RNA processing and modification
K	162	5.4	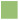	Transcription
L	124	4.6	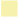	Replication, recombination, and repair
B	5	0.1	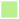	Chromatin structure and dynamics
D	31	0.8	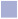	Cell cycle control, mitosis, and meiosis
Y	0	-	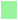	Nuclear structure
V	48	1.5	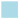	Defense mechanisms
T	43	3.4	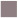	Signal transduction mechanisms
M	97	4.5	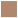	Cell wall/membrane biogenesis
N	19	0.6	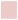	Cell motility
Z	3	0.1	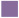	Cytoskeleton
W	0	-	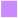	Extracellular structures
U	28	1.2	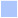	Intracellular trafficking and secretion
O	86	3.8	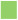	Posttranslational modification, protein turnover, chaperones
C	142	5.7	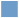	Energy production and conversion
G	163	7.1	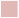	Carbohydrate transport and metabolism
E	236	11.8	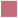	Amino acid transport and metabolism
F	76	3.4	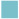	Nucleotide transport and metabolism
H	92	3.7	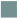	Coenzyme transport and metabolism
I	84	3.7	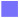	Lipid transport and metabolism
P	126	4.8	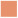	Inorganic ion transport and metabolism
Q	56	23	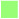	Secondary metabolite biosynthesis, transport, and catabolism
R	318	13.6	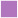	General function prediction only
S	314	9.5	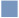	Function unknown
-	1181	35.1	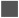	Not in COGs

* COG: Cluster of Orthologous Genes. COG color represents the corresponding color in Figure 1.

**Table 4:** Comparative analysis of genome between strains *D. irradiatisoli* 17bor-2, *D. radiodurans* R1^T^, and *D. geothermalis* DSM 11300^T^. The provided list enumerates the genes that play a crucial role in the pathways responsible for DNA repair and protein damage pathways. + and – symbols indicate the present and absent of the gene.

Subsystem	Gene Name	*Deinococcus irradiatisoli* 17bor-2	*Deinococcus radiodurans* R1^T^	*Deinococcus* *geothermalis* DSM 11300^T^
UvrABC system	Excinuclease ABC subunit A	+	+	+
	Excinuclease ABC subunit B	+	+	+
	Excinuclease ABC subunit C	+	+	+
Bacterial	A/G-specific adenine glycosylase	+	+	+
	DNA repair protein RadA	+	+	+
	DNA repair protein RecN	+	+	+
	Exodeoxyribonuclease III	+	+	+
	Exodeoxyribonuclease VII large subunit	+	+	+
	Exodeoxyribonuclease VII small subunit	+	+	+
	Exonuclease SbcC	+	+	+
	RecA protein	+	+	+
	Single-stranded DNA-binding protein	+	+	+
	Exonuclease SbcD	+	–	–
Bacterial MutL-MutS system	DNA mismatch repair protein MutL	+	+	+
	DNA mismatch repair protein MutS	+	+	+
	Recombination inhibitory protein MutS2	+	+	+
Bacterial RecBCD pathway	RecD-like DNA helicase YrrC	+	+	+
	ATP-dependent DNA helicase RecQ	+	+	+
	DNA recombination and repair protein RecF	+	+	+
	DNA recombination and repair protein RecO	+	+	+
	RecA protein	+	+	+
	Recombination protein RecR	+	+	+
	Single-stranded DNA-binding protein	+	+	+
	Single-stranded DNA-specific exonuclease RecJ	+	+	+
UvrD and related helicases	ATP-dependent DNA helicase UvrD/PcrA	+	+	–
RecA and MutS	DNA mismatch repair protein MutS	+	+	+
	RecA protein	+	+	+
Uracil-DNA glycosylase	G: T/U mismatch-specific uracil/thymine DNA-glycosylase	+	+	–
	Uracil-DNA glycosylase, family 1	+	+	–
	Uracil-DNA glycosylase, family 4	+	+	–

## Data Availability

The data used in study are available under the NCBI accession number CP029494.
